# Role of peptidylarginine deiminase 2 (PAD2) in mammary carcinoma cell migration

**DOI:** 10.1186/s12885-017-3354-x

**Published:** 2017-05-26

**Authors:** Sachi Horibata, Katherine E. Rogers, David Sadegh, Lynne J. Anguish, John L. McElwee, Pragya Shah, Paul R. Thompson, Scott A. Coonrod

**Affiliations:** 1 Baker Institute for Animal Health, College of Veterinary Medicine, Cornell University, Ithaca, NY 14850 USA; 2 Department of Molecular Medicine, College of Veterinary Medicine, Cornell University, Ithaca, NY 14850 USA; 3 Department of Biochemistry and Molecular Pharmacology, University of Massachusetts Medical School, Worcester, MA 01605 USA

**Keywords:** Peptidylarginine deiminase 2 (PAD2), Tumor cell migration, Ductal invasion, Mammary hyperbranching

## Abstract

**Background:**

Penetration of the mammary gland basement membrane by cancer cells is a crucial first step in tumor invasion. Using a mouse model of ductal carcinoma in situ, we previously found that inhibition of peptidylarginine deiminase 2 (PAD2, aka PADI2) activity appears to maintain basement membrane integrity in xenograft tumors. The goal of this investigation was to gain insight into the mechanisms by which PAD2 mediates this process.

**Methods:**

For our study, we modulated PAD2 activity in mammary ductal carcinoma cells by lentiviral shRNA-mediated depletion, lentiviral-mediated PAD2 overexpression, or PAD inhibition and explored the effects of these treatments on changes in cell migration and cell morphology. We also used these PAD2-modulated cells to test whether PAD2 may be required for EGF-induced cell migration. To determine how PAD2 might promote tumor cell migration in vivo, we tested the effects of PAD2 inhibition on the expression of several cell migration mediators in MCF10DCIS.com xenograft tumors. In addition, we tested the effect of PAD2 inhibition on EGF-induced ductal invasion and elongation in primary mouse mammary organoids. Lastly, using a transgenic mouse model, we investigated the effects of PAD2 overexpression on mammary gland development.

**Results:**

Our results indicate that PAD2 depletion or inhibition suppresses cell migration and alters the morphology of MCF10DCIS.com cells. In addition, we found that PAD2 depletion suppresses the expression of the cytoskeletal regulatory proteins RhoA, Rac1, and Cdc42 and also promotes a mesenchymal to epithelial-like transition in tumor cells with an associated increase in the cell adhesion marker, E-cadherin. Our mammary gland organoid study found that inhibition of PAD2 activity suppresses EGF-induced ductal invasion. In vivo, we found that PAD2 overexpression causes hyperbranching in the developing mammary gland.

**Conclusion:**

Together, these results suggest that PAD2 plays a critical role in breast cancer cell migration. Our findings that EGF treatment increases protein citrullination and that PAD2 inhibition blocks EGF-induced cell migration suggest that PAD2 likely functions within the EGF signaling pathway to mediate cell migration.

**Electronic supplementary material:**

The online version of this article (doi:10.1186/s12885-017-3354-x) contains supplementary material, which is available to authorized users.

## Background

Cell migration is a highly complex process that is involved in various biological processes ranging from embryonic development to the immune response [1–3]. Moreover, it is often dysregulated in cancer and plays an important role in metastasis and invasion [4, 5]. To migrate, cells modify their shape and stiffness by becoming polarized and elongated [6], forming protrusions and extensions which branch outwards to define the leading edge [7, 8]. These protrusions are functionally diverse and include lamelipodia, filopodia, spikes (early filopods), pseudopods, ruffles (early pseudopods), or invadopods [9, 10]. While the newly formed leading edge establishes the formation of new adhesion sites [11], changes in the trailing edge are also required for cells to move forward. For example, cells must lose contact with the previous adhesion site at the trailing edge and, when contraction of the actomyosin cytoskeleton occurs, the trailing edge must generate sufficient traction force to allow the cell body to move forward [4, 12].

Given the critical role of cell migration in biological processes, it is not surprising that its dysregulation is associated with cancer progression. In normal breasts, there are two distinct cell layers that line the mammary duct. A basal layer of myoepithelial cells is bounded by a basement membrane that separates the mammary gland epithelium from the surrounding stromal tissues. Superficial to the basal layer are the luminal epithelial cells [13]. In luminal breast cancers, the luminal epithelial cells proliferate uncontrollably and fill the mammary gland, thereby disrupting tissue architecture and eventually breaking the myoepithelial basement membrane. Once free from the basement membrane, the cancer cells can then migrate out from the mammary gland, resulting in invasive breast cancer [14]. It is thought that ductal carcinoma in situ (DCIS) lesions become invasive when the tumor cells undergo an epithelial-to-mesenchymal transition (EMT), thus facilitating their migration across the basement membrane [15, 16]. Cancer cells undergoing EMT frequently downregulate E-cadherin leading to a loss of epithelial cell polarity and adhesiveness [17] and these tumor cells also acquire mesenchymal cell characteristics that include increased cell motility and invasiveness. Multiple signaling pathways have been found to regulate EMT including TGF-β, PI3K/AKT, and the transcription factors Snail, Slug, and Smad [16, 18–20].

Initiation of cell migration is often mediated by growth factors, chemokines and/or by components of the extracellular matrix (ECM) [4]. Epidermal growth factor (EGF) is a major inducer of cell migration in breast cancer cells and functions in this capacity by activating a phosphatidyl-inositol 3-kinase (PI3K) signal transduction cascade which then leads to the activation of the Rho family of small GTPases (including Rho, Rac, and Cdc42) [7, 10, 21–23]. These GTPases are regulated in a very specific manner in order to coordinate the proper movement of the cellular actin cytoskeleton [24]. Rac localizes to the leading edge where it promotes the formation of cell protrusions via coordination of actin polymerization. On the other hand, Rho localizes to the trailing edge where it helps coordinate the contraction of the actin cytoskeleton. Lastly, Cdc42 is involved in orienting the microtubule assembly in the direction of cell movement. The identity of other molecules that modulate Rho family-mediated cell migration remains unclear.

Peptidylarginine deiminases (PADs) are a family of calcium-dependent enzymes that post-translationally convert arginine residues into neutrally charged citrulline. This activity, referred to as citrullination or deimination, can alter the target protein’s tertiary structure or the substrate-binding of the target protein [25]. There are five PAD isozymes, PAD1–4, and 6, with each having a unique tissue distribution. Previously, we found that peptidylarginine deiminase 4 (PAD4) suppressed breast cancer cell migration via its role in modulating EMT [26]. Tanikawa et al. have also shown that ectopic expression of PAD4 results in inhibition of tumor cell growth [27]. Aside from PAD4, we also found that PAD2 localizes to mammary tissues and is dysregulated in breast cancer [28–31]. Unlike PAD4, we observed that PAD2 seems to be involved in promoting breast cancer progression [30, 32]. Using the pan-PAD inhibitor, Cl-Amidine (first generation inhibitor) [33–35], we recently found that the mammary duct basement membrane of MCF10DCIS.com xenograft tumor restores its basement membrane integrity [30]. To build upon on our previous findings, we used Cl-Amidine, as well as a more potent PAD inhibitor, BB-Cl-Amidine (second generation inhibitor) [36], to investigate the potential mechanisms by which PAD2 mediates tumor cell migration across the basement membrane, thereby enhancing tumor cell invasion.

## Methods

### Materials

The following antibodies were used: anti-PAD2 (12110–1-AP, Proteintech), anti-E-cadherin (ab1518, Abcam), anti-RhoA (2117, Cell Signaling), anti-Rac1 (05–389, Millipore), anti-Cdc42 (07–1466, Millipore), anti-pan-Citrulline (07–377, Millipore; ab6464, Abcam), and anti-β-actin (ab8227, Abcam) antibodies. PAD inhibitors, Cl-Amidine [33, 35] and BB-Cl-Amidine [36], were generous gifts from Dr. Paul Thompson (University of Massachusetts Medical School).

### Cell culture and lentivirus generation

Human comedo ductal carcinoma in situ cells (MCF10DCIS.com) were obtained from Dr. Fred Miller (Barbara Ann Karmanos Cancer Institute, Detroit, MI, USA). Generation of MCF10DCIS.com cells has been described [37]. Cells were cultured in DMEM/F-12 medium containing 5% horse serum (HS) and 1% penicillin streptomycin (P/S). To generate the *PADI2* knockdown and control cell lines, MCF10DCIS.com cells were stably transduced with lentivirus expressing shRNA (Mission shRNA from Sigma) for *PADI2* (TRCN0000051447 – NM_007365.1-995s1c1) or scrambled non-mammalian shRNA control (SHC002). Lentivirus was prepared and transduced as previously described in Campeau et al. using 3rd generation lentiviral packaging/envelope vectors (pLP1-pMDLg/pRRE [12,251, Addgene], pLP2-pRSV-Rev. [12,253, Addgene], and pVSV-G-pMD2.G [12,259, Addgene]) [38]. Selection of the stable clones was accomplished via treatment with 2 μg/ml of puromycin for at least 2 weeks. Scrambled control shRNA and *PADI2*-shRNA of MCF10DCIS.com cells were grown in the described MCF10DCIS.com media with addition of 2 μg/ml of puromycin.

### Immunoblot assay

Whole cell lysates (50 μg of protein per lane) were resolved by SDS-PAGE followed by transfer to PVDF membrane. The membranes were blocked in either 5% milk (for anti-E-cadherin, anti-RhoA, anti-Rac1, anti-Cdc42, and anti-β-actin) or 3% BSA (for anti-PAD2) at room temperature and were incubated overnight with primary antibodies diluted in TBST at 4 °C using the following antibody concentrations: anti-PAD2 (1:1000), anti-E-cadherin (1:1000), anti-RhoA (1:1000), anti-Rac1 (1:1000), and anti-Cdc42 (1:1000) antibodies. To ensure equal loading, membranes were probed with anti-β-actin (1:6000) antibody. The primary antibodies were detected with HRP-conjugated secondary antibodies and were exposed to ECL reagents.

### RNA isolation and quantitative real-time PCR

RNA was purified using RNeasy Kit (Qiagen) and reverse-transcribed using High Capacity RNA-to-cDNA kit (Applied Biosystems) according to the manufacturers’ protocols. Real-time quantitative PCR analysis was performed using the following primers [39, 40]: PAD2 (*PADI2*) Forward (5′-TCTCAGGCCTGGTCTCCA-3′) and Reverse (5′- AAGATGGGAGTCAGGGGAAT-3′); E-cadherin (*CDH1*) Forward (5′- TGGAGGAATTCTTGCTTTGC-3′) and Reverse (5′- CGCTCTCCTCCGAAGAAAC-3′); β-actin (*ACTB*) Forward (5′- CCAACCGCGAGAAGATGA-3′) and Reverse (5′- CCAGAGGCGTACAGGGATAG-3′); RhoA (*RHOA*) Forward (5′-CCAAATGTGCCCATCATCCTAGTTG-3′) and Reverse (5′-TCCGTCTTTGGTCTTTGCTGAACAC-3′); Rac1 (*RAC1*) Forward (5′-CAATGCGTTCCCTGGAGAGTACA-3′) and Reverse (5′-ACGTCTGTTTGCGGGTAGGAGAG-3′); Cdc42 (*CDC42*) Forward (5′-TAACTCACCACTGTCCAAAGACTCC-3′) and Reverse (5′-CCTCATCAAACACATTCTTCAGACC-3′) and Power SYBR Green PCR Master Mix (Applied Bioystems). The samples were normalized to β-actin and three biological replicates were performed. The Applied Biosystems StepOne Real-Time PCR System was used to perform this assay.

### Immunofluorescence (IF) and immunohistochemistry (IHC)

Cells were fixed with 4% paraformaldehyde for 15 min and were permeabilized and blocked with PBS containing 0.1% Triton X-100 and 10% BSA. Cells were incubated overnight with indicated primary antibodies in 1:100 dilutions at 4 °C. Cells were rinsed with PBS and were incubated with secondary antibody diluted in PBS containing DAPI (1:50,000) for 1 h at room temperature. Cells were washed with PBS and mounted using ProLong antifade mounting reagent (P36934; Life Technologies). IF and IHC of tissue sections were prepared using a standard protocol as described previously [41].

### Cell migration (scratch or wound healing assays)

MCF10DCIS.com cells expressing scrambled shRNA or *PADI2*-shRNA were grown to confluence in their regular selection medium (DMEM/F-12 medium containing 5% HS, 1% P/S, and 2 μg/ml). A wound was generated on a cover slip using a pipette tip and the culture media was changed to remove detached cells. Eighteen or twenty-four hours later, the cells were fixed for 12 min with 4% paraformaldehyde, washed with PBS, and visualized by light microscopy. When examining the effect of EGF or BB-Cl-Amidine on cell migration, the MCF10DCIS.com cells were first grown in their regular selection media for plating and then serum starved for 24 h. Once the cells were confluent, a wound was struck and the detached cells were removed. DMEM/F-12 containing the following treatments were added: control (DMSO), EGF (100 ng/ml), BB-Cl-Amidine (0.5 μM), or both EGF (100 ng/ml) and BB-Cl-Amidine (0.5 μM). For live cell-imaging of cell migration, after the wound was struck and the detached cells were removed, cells were imaged every 10 min over the duration of 24 h.

### Live cell imaging to track single or multi cell migration assay

Scrambled shRNA and *PADI2*-shRNA MCF10DCIS.com cells were plated lightly (~10–20% confluence) in tissue culture flask and cultured overnight in cell culture incubator. Once the cells adhered to the bottom of the flask, the flask containing the cells was transferred to the Zeiss Axio Observer inverted microscope with built-in incubator. The Axiovision software was used to image the cells every 10 min for the duration of 15 or 19 h. For the single cell migration assay, 20 cells per group (*n* = 20) were monitored using the Tracking Tool™ Pro v2.0.

### Generation of MCF10DCIS.Com xenograft mice

MCF10DCIS.com xenograft mice were generated and treated with PBS or Cl-Amidine (50 mg/kg/day) using the protocol described previously [30]. In here, Cl-Amidine was used as we have optimized the use of Cl-Amidine for in vivo studies.

### Mammary branching in wild-type and PAD2-overexpressing mice

Wild type (FVB) and PAD2-overexpressing (MMTV-PAD2) 8 week old female mice were used to examine differences in mammary gland development under the influence of increased PAD2 expression in vivo. The derivation of MMTV-PAD2 mice has been described previously [39]. The estrous cycle of mice was determined by vaginal flushes and cytological examination and only those in diestrous were used for mammary harvest. Mice were sacrificed by CO_2_ inhalation and both mammary glands were removed as described by Plante et al. [42]. One gland was fixed in 10% neutral buffered formalin, processed and embedded in paraffin for sectioning. Five micron sections were stained with hematoxalin and eosin (H&E). The other gland was fixed in Carnoy’s, stained with carmine alum, and whole mounted on a slide [42] for measurement of ductal elongation and alveolar branching. Images using 5× (low magnification) and 20× (high magnification) objectives of H&E stained slides were obtained using Axiovision software on a Zeiss Axio Observer inverted microscope equipped with an Axiocam color CCD camera. To measure branching, whole mounts were imaged using 0.8× and 5× objectives and branching was quantified on images using ImageJ software by counting the number of branching points (nodes) in three different and defined areas of 250 X 150 pixels for each tissue sample. Three mice were used for each group. All experimental procedures using mice followed recommended guidelines and were approved by the Institutional Animal Care and Use Committee at Cornell University.

### Ductal invasion and elongation in primary mouse mammary organoids

Primary mouse mammary organoids were prepared using the protocol described previously [43]. Eight week old female mice were sacrificed and their mammary glands were isolated. Isolated mammary glands were cut and digested using digestion buffer (2 g/l trypsin, 2 g/l collagenase type-iv, 5% [*v*/v] FBS, 5 μg/ml insulin in DMEM/F12 medium) and incubated on a shaker at 37 °C rotating at 100 rpm for 30 min. Following centrifugation, organoids were collected from the bottom layer, purified, and enriched. Organoids were then embedded in matrigel in 8 well-tissue slide chambers. Twenty four hours later, the samples were treated either with control (DMSO), EGF (100 ng/ml), EGF (100 ng/ml) + BB-Cl-Amidine (0.5 μM), or EGF (100 ng/ml) + BB-Cl-Amidine (5 μM). The media (along with specific treatment doses) were replaced every 3 days. The samples were imaged using Zeiss Axio Observer inverted microscope after 6 days. Three mice (*n* = 3) were used for each treatment. For this particular assay, we used the next-generation PAD inhibitor, BB-Cl-Amidine, to test whether PAD2 plays a role in ductal invasion.

### Statistical analysis

Statistical analyses were performed using GraphPad Prism 7. Two groups were compared using two-tailed Student’s t-test. Sample sizes (n) and statistical significance (*p*-values) are indicated in the Figure Legends.

## Results

### PAD2 promotes breast cancer cell migration.

**Fig. 1:**
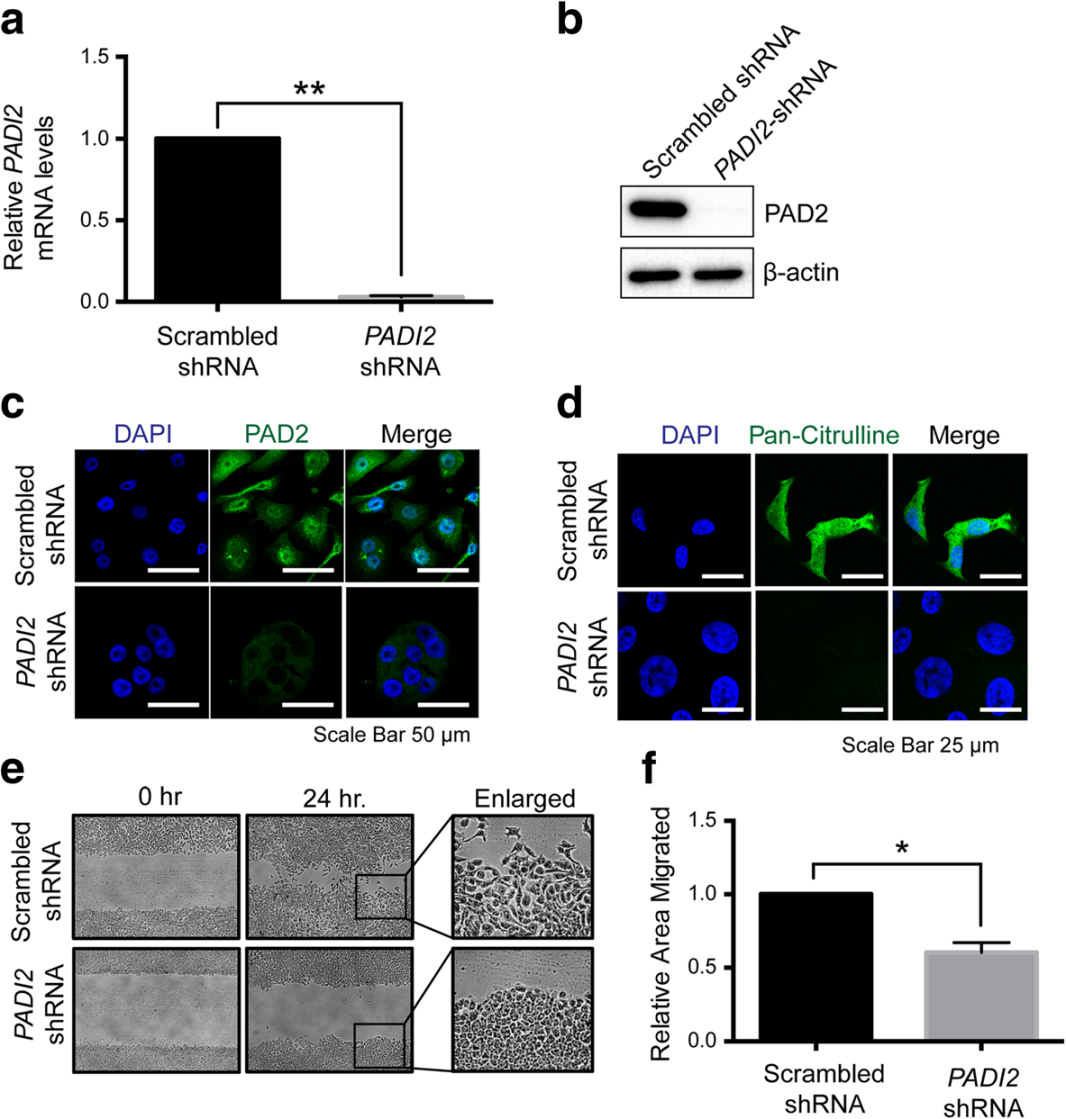
Depletion of PAD2 suppresses cell migration in MCF10DCIS.com cells. **a** Total RNA was isolated from MCF10DCIS.com cells infected with scrambled-shRNA and *PADI2*-shRNA lentiviruses. *PADI2* mRNA levels were determined by qRT-PCR (SYBR) using scrambled-shRNA as a reference and β-actin normalization. Data were analyzed using the 2 ^-ΔΔ C(t)^ method and are expressed as the mean ± SD from three independent experiments (*p < 0.05). **b** The whole cell lysates of MCF10DCIS.com scrambled-shRNA and *PADI2*-shRNA cells were immunoblotted with anti-PAD2 antibody. Anti-β-actin was used as loading control. **c** Immunofluorescent analysis was performed on scrambled-shRNA and *PADI2*-shRNA cells probed with anti-PAD2 antibody, (**d**) anti-pan-citrulline antibody, and DAPI (nuclei) staining. Scale bars = (C) 50 μm and (D) 25 μm. **e** Relative average areas of wound closure from the wound healing assays were calculated using ImageJ and normalizing to scrambled-shRNA (**p < 0.05). **f** A wound healing assays were performed on MCF10DCIS.com scrambled-shRNA and *PADI2*-shRNA cells. The cells were fixed using 4% paraformaldehyde 24 h after striking the wound (24 h.). The cells were then visualized and imaged using light microscopy to determine the extent of the wound closure. The widths of the initial wounds are indicated by the lines. The cells were also fixed immediately after striking the wound (0 h.) to indicate the size of the initial wounds. The images on the right are the enlarged images of the boxed images

To investigate whether PAD2 promotes the migration of breast cancer cells, we first generated PAD2-depleted ductal carcinoma in situ (MCF10DCIS.com) cells using the lentiviral shRNA knockdown system. We confirmed *PADI2-*knockdown using quantitative real time PCR and found that the PAD2-depleted line showed a 97% reduction in *PADI2* mRNA levels when compared to the scrambled shRNA cell line (Fig. 1a). Furthermore, immunoblot and immunofluorescent analyses demonstrated that PAD2 protein levels were also reduced in the PAD2-depleted line (Fig. 1b and c). Additionally, immunofluorescent analysis revealed that global levels of citrullinated proteins were reduced in the PAD2-depleted line compared to the shRNA control line (Fig. 1d).

We then tested the effects of PAD2 depletion on cell migration. Using a wound healing assay, we found that depletion of PAD2 in MCF10DCIS.com cells inhibited cell migration, with a 39% reduction in migration in the PAD2-depleted cells compared to the scrambled shRNA cell line (Fig. 1e and f). To further test the requirement of PAD2 for cell invasion, we also investigated the migratory potential of PAD2 overexpressing MCF10AT cells using the wound healing assay. Results showed that the migratory potential of PAD2 overexpressing cells was significantly greater than that of the control cell line (Additional file 1: Fig. S2).

Interestingly, while quantifying the effects of PAD2 on cell migration, we observed that the PAD2-depleted cells appeared to be more rounded and tightly packed when compared to the control group, which contained elongated cells that had frequently appeared to migrate independently into the wound (Fig. 1f). This observation suggested that PAD2 may be required for early metastatic events where epithelial cells lose their cell-cell adhesion properties and gain mesenchymal properties to become more migratory [17].

**Fig. 2:**
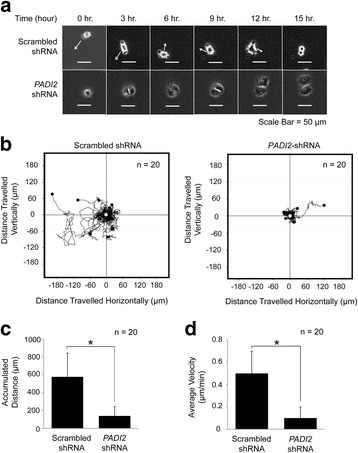
Single cell migration is impaired in PAD2 depleted MCF10DCIS.com cells. **a** Images of MCF10DCIS.com scrambled-shRNA and *PADI2*-shRNA cells taken at times 0, 3, 6, 9, 12, and 15 h at the same location using a wide-field microscope with a built-in incubation chamber. The arrow indicates the directional movement of the cells. Scale bar = 50 μm. **b** Graphical representation of the movement of individual cells (*n* = 20) of scrambled-shRNA and *PADI2*-shRNA cells in culture. The line shows the trails of the cell movement and the black circle shows the final location of the cells. The point of origin is shown as a white dot. Graphs were generated using Tracking Tool™ Pro v2.0. **c** Graph depicts accumulated distance travelled and (**d**) average velocity by single scrambled-shRNA and *PADI2*-shRNA cells

We also noted that PAD2 depletion results in reduced cell proliferation (Additional file 2: Fig. S1). To test whether the migratory phenotype seen in Fig. 1e was caused by cell movement and not by cell proliferation, we tracked the movement of single cells using time-lapse imaging. Results showed that the PAD2-depleted cells appeared to be actively dividing; however, the resulting daughter cells displayed diminished mobility (Fig. 2a). On the other hand, the shRNA control cells were more migratory and more dynamic in cell shape.

We further analyzed 20 shRNA control and 20 PAD2-depleted cells during the course of 19 h and tracked the distance travelled away from the point of origin. Strikingly, most of the PAD2-depleted cells remained stationary, while the control cells actively migrated away from the point of origin (Fig. 2b. Using the Tracking Tool™ Pro v2.0, we quantified the accumulated distance travelled by these cells and found that the control cells had an average total distance travelled of 577.0 μm whereas the PAD2-depleted cells only travelled 139.1 μm (Fig. 2c). Furthermore, we found the average velocity of the control cells to be 0.5 μm/min, with the maximum velocity of 1.3 μm/min. In contrast, the PAD2-depleted cells had an average velocity of 0.1 μm/min, with the maximum velocity of 0.3 μm/min (Fig. 2d). This represents a 5-fold reduction in average velocity in the PAD2-depleted cells compared to the control cells. These results suggest that PAD2 plays an important role in regulating cell migration.

### Depletion of PAD2 reduces cell protrusion and downregulates cytoskeletal markers

**Fig. 3:**
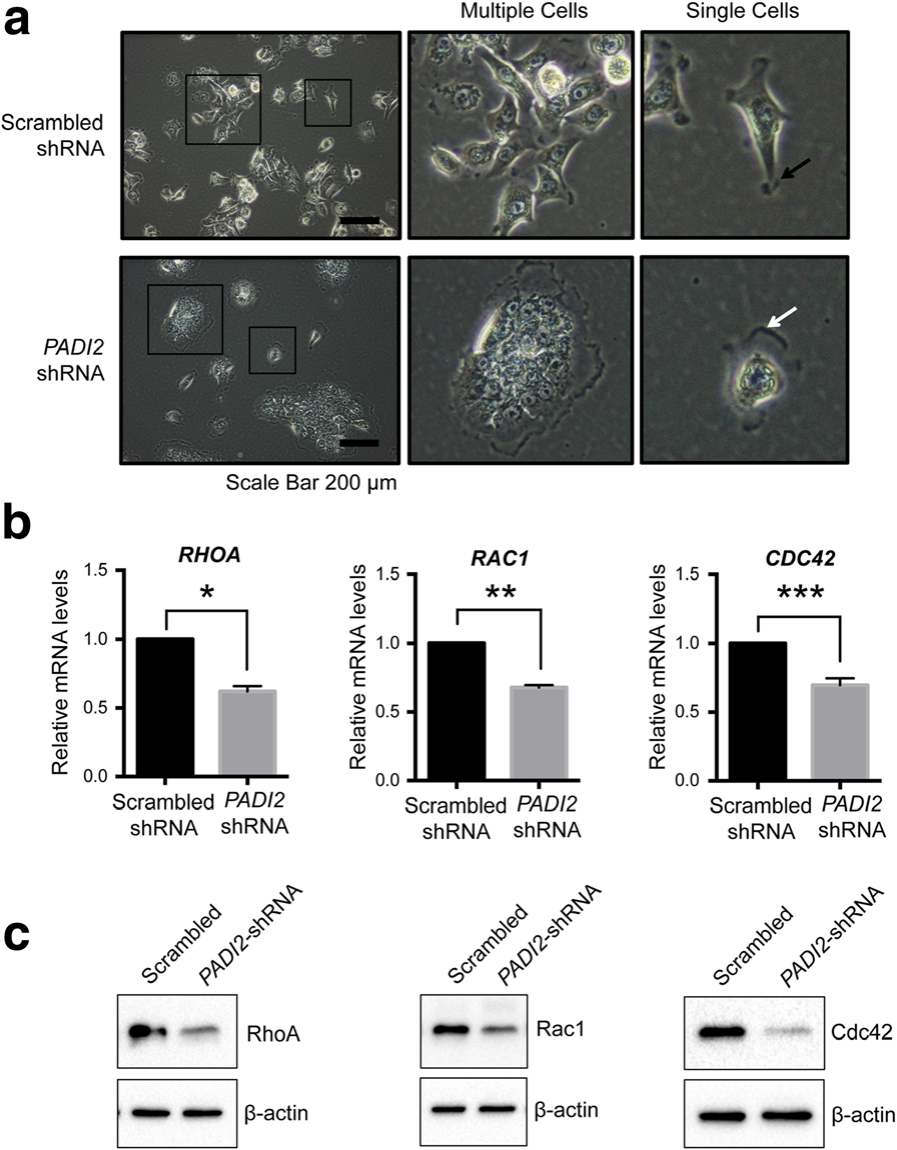
PAD2 depletion alters the morphology of MCF10DCIS.com cells. **a** Representative images of scrambled-shRNA and *PADI2*-shRNA cells in culture. Scale bar = 200 μm. The frames on the images to the left are enlarged on the middle and right images. The middle images show the morphology of multiple cells that are growing together; images on the right are the pictures of single cells. The black arrow indicates cell protrusion and membrane ruffling in the scrambled-shRNA cells. The white arrow shows the flattened appearance of the cytoplasmic region of *PADI2*-shRNA cells. **b** Total RNA was isolated from scrambled-shRNA and *PADI2*-shRNA cells. *RHOA, RAC1,* and *CDC42* mRNA levels were determined by qRT-PCR (SYBR) using scrambled-shRNA as a reference and β-actin normalization. Data were analyzed using the 2 ^-ΔΔ C(t)^ method and are expressed as the mean ± SD from five independent biological replicates with three technical replicates for each biological replicate (* *p* < 0.05). **c** The whole cell lysates of MCF10DCIS.com scrambled-shRNA and *PADI2*-shRNA cells were immunoblotted with RhoA, Rac1, and Cdc42. The blot was also probed with anti-β-actin as a loading control

Cells migrate by altering their shape and stiffness leading to a polarized and elongated phenotype [6]. We next tested whether PAD2 depletion alters the morphological changes that are required for cell migration. We found that PAD2-depleted cells are less elongated and appeared flatter in shape and more tightly adhered to the underlying plate (Fig. 3a, *white arrow*). Additionally, they were predominantly grouped together rather than isolated as single cells, again, suggesting that these cells may be more adherent. In contrast, the control cells were elongated in shape with membrane ruffling at the edge of the cell protrusions (Fig. 3a, *black arrow*). In addition, we observed cells in the control group with up to five protrusions, thus highlighting their dynamic movement. Furthermore, we found enlarged nuclei in single, isolated PAD2-depleted cells compared to control cells (Fig. 3a). However, when the cells were grouped or adhered to other cells, the nuclei of PAD2-depleted cells were smaller in size.

Next, we tested whether the distinct changes in cell morphology caused by PAD2 depletion were associated with cytoskeletal changes. More specifically, we tested for expression levels of the Rho family of GTPases, RhoA, Rac1, and Cdc42, as these actin cytoskeleton markers are known to regulate cell migration [24]. Interestingly, we found that there were significant reductions in *RHOA* (38.0%), *RAC* (32.1%)*,* and *CDC42* (30.3%) transcript levels in the PAD2-depleted cells compared to the control line (Fig. 3b). Furthermore, immunoblot assays confirmed our mRNA findings (Fig. 3c). Collectively, these results suggest that PAD2 promotes cell migration by modulating the cytoskeletal machinery that is required for cell motility.

### Cell adhesion increases upon PAD2 depletion

**Fig. 4:**
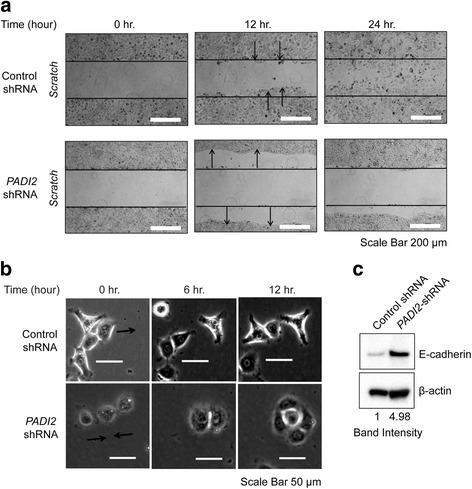
Enhanced cell-cell adhesion is observed in *PADI2*-depleted cells. **a** Scratch assays were performed on scrambled-shRNA and *PADI2*-shRNA cells. The cells were visualized and imaged at *t* = 0, 12, and 24 h, using a wide-field microscope with built-in incubation chamber, to determine the extent of the wound closure. Widths of initial wounds are indicated by lines. Arrow indicates the directional movement of the migrating cells. Scale bar = 200 μm. **b** Images of 2 to 3 scrambled-shRNA and *PADI2*-shRNA cells were taken at *t* = 0, 6, and 12 h. Scale bar = 50 μm. **c** Whole cell lysates from scrambled-shRNA and *PADI2*-shRNA cells were immunoblotted with anti-E-cadherin antibody. Anti-β-actin was used as a loading control. The intensity of the band was measured using ImageJ and was normalized to scrambled-shRNA cells

Aside from changes in cell morphology, we also observed changes in the adhesive properties of PAD2-depleted cells. During the time course of the wound healing assay, we found that control cells migrated into the wound following the initial scratch as described previously. Surprisingly, in stark contrast, PAD2-depleted cells appeared to initially contract away from the wound before eventually filling the vacated area up to the point of the scratch (Fig. 4a). We next investigated the adhesive properties of single cells. In PAD2-depleted cells, we found that, over time, independent cells would adhere to each other eventually forming cell clusters (Fig. 4b). In contrast, we found that the control cells would often detach from each other and migrate independently. These observations suggest that PAD2 depletion may lead to the upregulation of cell-cell adhesion molecules, such as E-cadherin. We tested this hypothesis and found that depletion of PAD2 upregulates the expression of E-cadherin by approximately 5-fold (Fig. 4c). Together, these findings suggest that depletion of PAD2 suppresses cell migration by promoting the upregulation of factors that are involved in cell-cell adhesion.

### PAD2 may function within the EGF signaling pathway to regulate cell migration

**Fig. 5:**
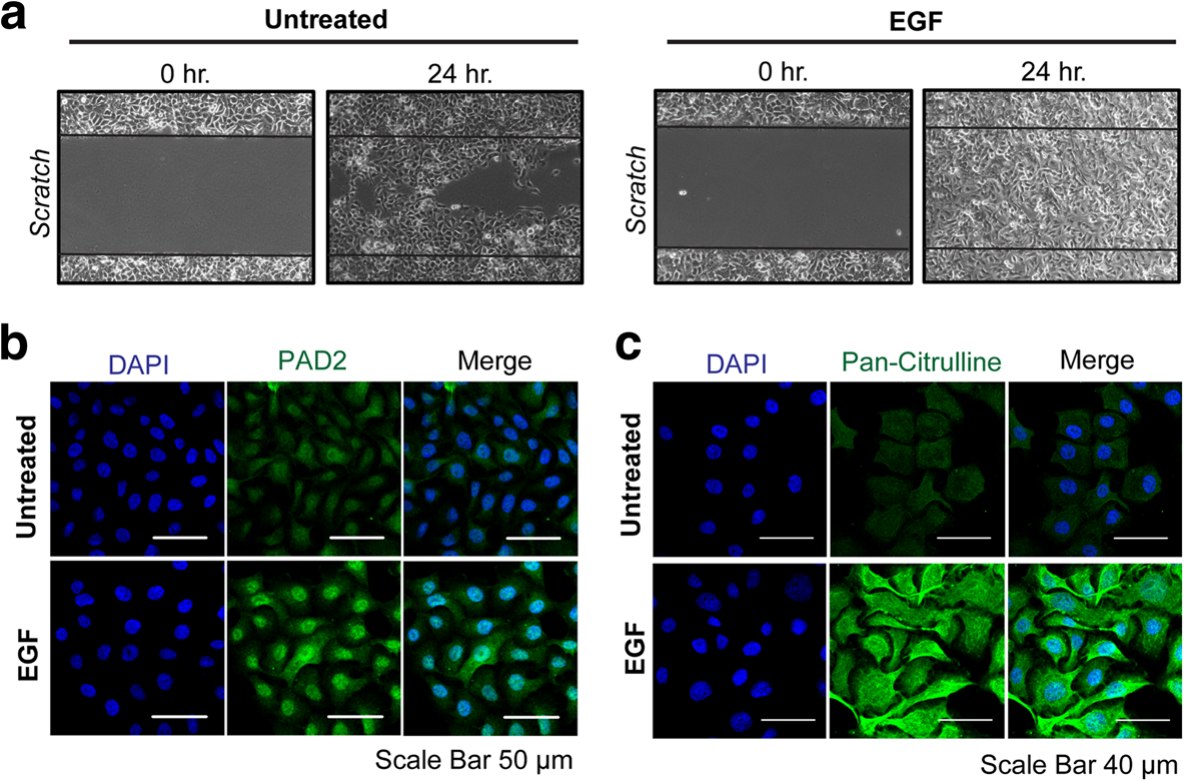
Enhanced activity and expression of PAD2 observed in EGF treated MCF10DCIS.com cells. **a** Scratch assays were performed on serum-deprived MCF10DCIS.com cells treated without (*Untreated*) or with EGF. MCF10DCIS.com cells were fixed 24 h after wound striking. Cells were visualized using light microcopy to determine the extent of wound closure. Serum-deprived MCF10DCIS.com cells were treated without (*Untreated*) or with EGF for 1 h prior to immunofluorescent analysis using (**b**) anti-PAD2 and (**c**) anti-pan-citrulline antibodies. DAPI was used to visualize the nucleus. Scale bar = 50 μm

Given that EGF signaling plays an important role in cancer cell polarization and migration, we tested whether EGF may regulate PAD2 expression and activity and whether modulation of PAD2 activity may affect EGF-induced cell migration. We first tested the ability of EGF to induce cell migration in MCF10DCIS.com cells and observed robust enhancement of cell migration into the wound following EGF treatment, thus confirming that EGF promotes the migration of MCF10DCIS.com cells (Fig. 5a). We then tested the effect of EGF treatment on PAD2 expression and activity. Immunofluorescent analysis found that treatment of MCF10DCIS.com cells with EGF for 60 min strongly increased PAD2 expression and activity in these cells (Fig. 5b and c). Interestingly, we found that EGF treatment resulted in a strong increase in nuclear PAD2 levels. Together, these suggest that PAD2 may function within the EGF signaling pathway to regulate cell migration.

### PAD inhibitor, BB-cl-Amidine, suppresses EGF-induced cell migration

**Fig. 6:**
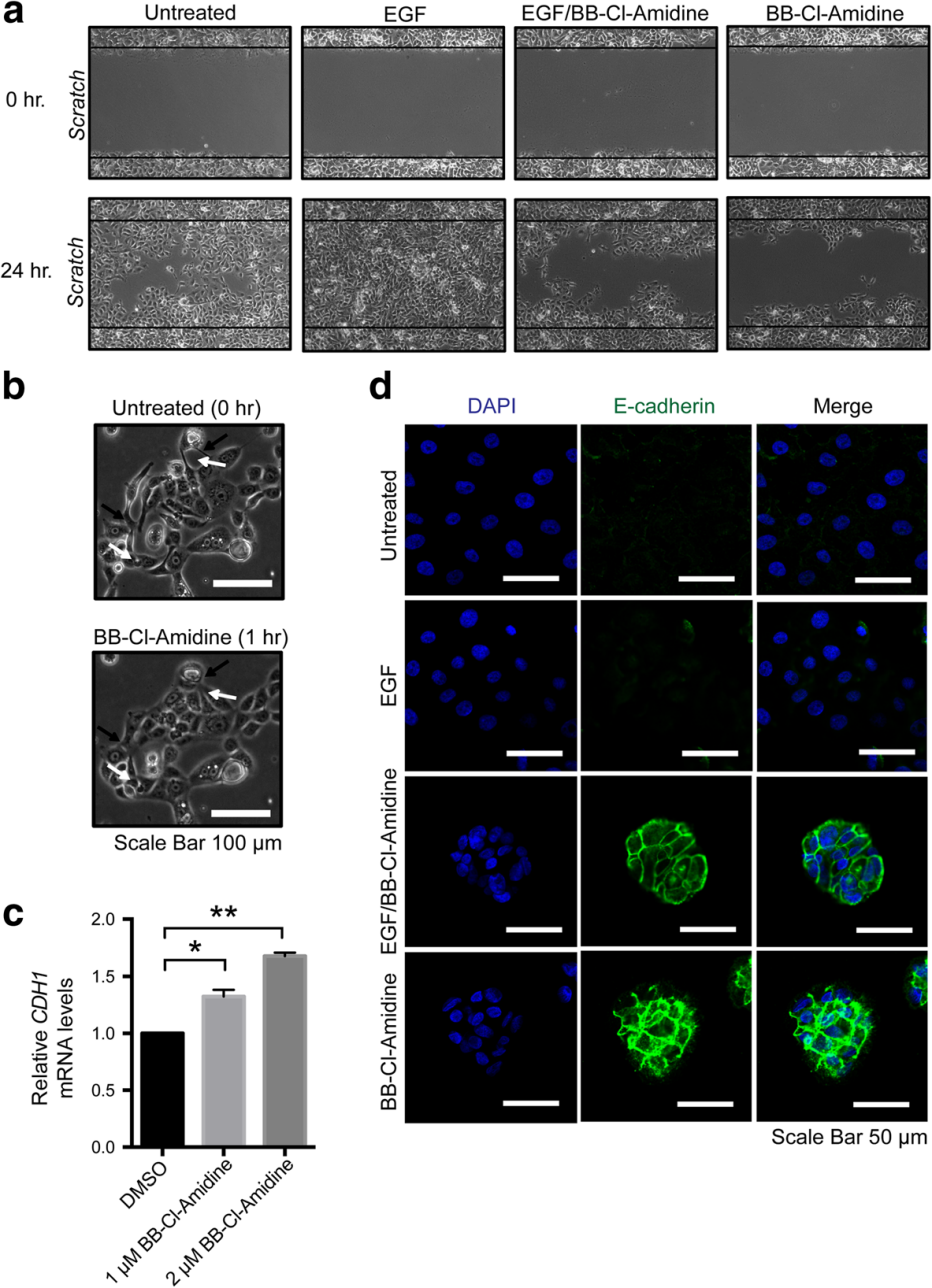
BB-Cl-Amidine treatment suppresses cell migration and enhances cell adhesion in MCF10DCIS.com cells. **a** Scratch assays were performed on serum-deprived MCF10DCIS.com cells following either no treatment (*Untreated*) or treatment with EGF, EGF + BB-Cl-Amidine, or with BB-Cl-Amidine alone. MCF10DCIS.com cells were pre-treated with BB-Cl-Amidine for 23 h and then EGF was added for 1 h. Cells were fixed using 4% paraformaldehyde 24 h after striking the wound (24 h) and then visualized and imaged using light microscopy to determine the extent of the wound closure. Widths of initial wounds are indicated by the lines. Cells were fixed immediately after striking the wound (0 h) to indicate the size of the initial wounds. **b** MCF10DCIS.com cells were treated either with DMSO or 2 μM BB-Cl-Amidine. One hour after treatment, the cells were imaged using light microscopy. Scale bar = 100 μm. **c** Total RNA was isolated from MCF10DCIS.com cells treated with either 0 (DMSO), 1 μM, or 2 μM BB-Cl-Amidine. *CDH1* mRNA levels were determined by qRT-PCR (SYBR) using DMSO treated control cells as a reference and β-actin for normalization. Data were analyzed using the 2 ^-ΔΔ C(t)^ method and are expressed as the mean ± SD from two biological replicates with three technical replicates per biological replicate (* *p* < 0.05; ** *p* < 0.005). **d** Anti-E-cadherin immunofluorescence staining was performed on serum-deprived MCF10DCIS.com cells following either no treatment (*Untreated*) or treatment with EGF, EGF + BB-Cl-Amidine, or with BB-Cl-Amidine alone. MCF10DCIS.com cells were pre-treated with BB-Cl-Amidine for 23 h and then EGF was added for 1 h. Scale bar = 50 μm

The finding that EGF appears to upregulate PAD2 expression and activity suggested that PAD2 may play a role in EGF-mediated cell migration. We tested this hypothesis and found that the PAD inhibitor, BB-Cl-Amidine [36], suppressed EGF-induced migration of MCF10DCIS.com cells (Fig. 6a). Interestingly, we also found that when PAD2 was depleted from cells, EGF treatment did not promote cell migration (Additional file 3: Fig. S3). Importantly, we found that BB-Cl-Amidine treatment promoted the conversion of MCF10DCIS.com cells from an elongated fibroblast-like migratory phenotype to a more epithelial-like adherent phenotype which is similar to that of the PAD2-depleted cells (Fig. 6b).

We then tested whether BB-Cl-Amidine treatment increased the expression of the cell-cell adhesion molecule, E-cadherin, in MCF10DCIS.com cells. We found that MCF10DCIS.com cells expressed significantly higher levels of *CDH1* mRNA levels after treatment with BB-Cl-Amidine (Fig. 6c). Immunofluorescence analysis supported our qRT-PCR results as we found that E-cadherin levels appeared to be higher in the BB-Cl-Amidine treated cells than control cells (Fig. 6d). The increase was also observed in cells treated with BB-Cl-Amidine in the presence of EGF. As seen by the DAPI staining, the cells are in closer proximity to each other in the presence of BB-Cl-Amidine, further suggesting increased adhesion when PAD2 activity is inhibited. These results suggest that one mechanism by which PAD2 activity promotes cell migration is by downregulating the expression of cell-cell adhesion molecules in an EGF-dependent manner.

### Cl-Amidine treatment increases E-cadherin expression level in vivo

**Fig. 7:**
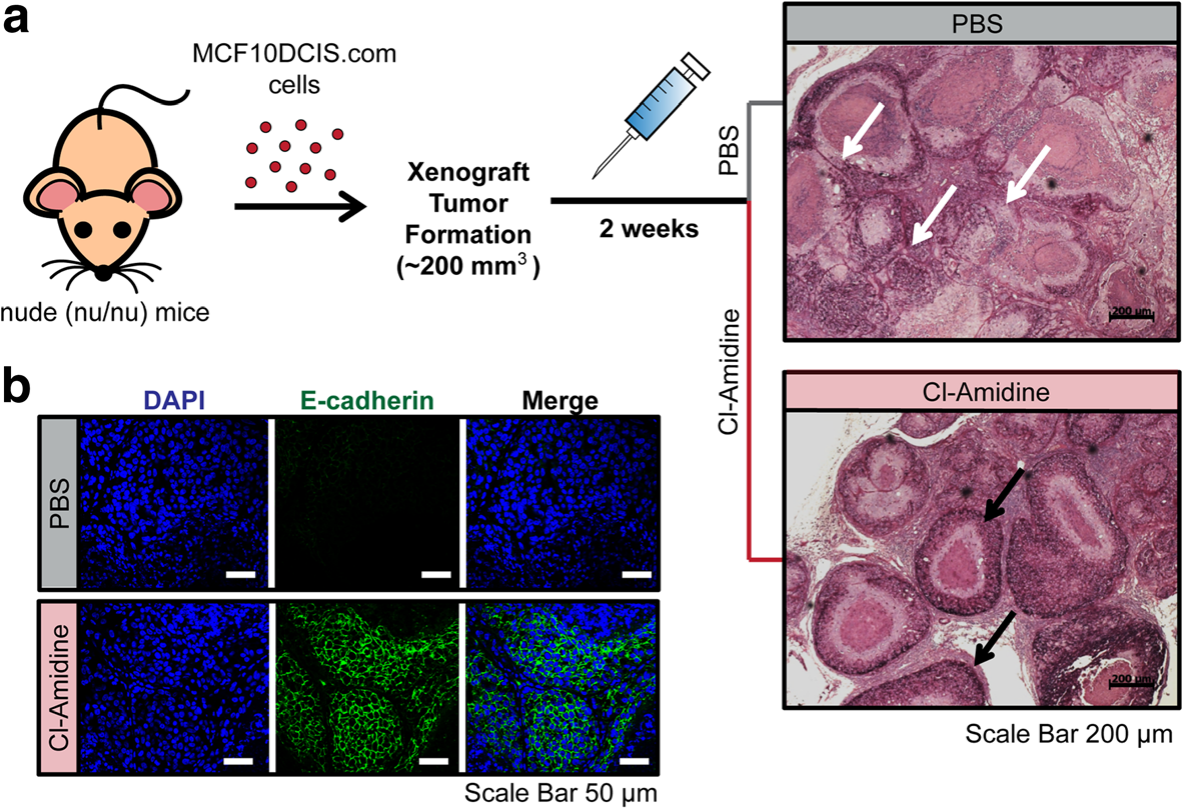
Cl-Amidine treatment increases E-cadherin expression in MCF10DCIS.com xenograft tumors. **a** PAS staining of representative mammary tissue sections of MCF10DCIS.com xenograft mice following either vehicle alone treatment (PBS) or treatment with Cl-Amidine. PAS stained sections were prepared and imaged using the bright field optics of the Axiophot inverted microscope. Scale bar = 200 μm. **b** Anti-E-cadherin IF staining of tissue sections from MCF10DCIS.com xenograft tumors following vehicle alone treatment (PBS) or with Cl-Amidine treatment. Scale bar = 50 μm

Previously, we generated a DCIS mouse xenograft model and tested the effects of the first-generation PAD inhibitor, Cl-Amidine, on tumor growth [30]. In this model system we consistently found that tumor cells in the Cl-Amidine treated mice appeared to be less invasive and that the basement membranes of the ducts within the treated tumors were more intact (Fig. 7a). Therefore, we used this model system to test whether PAD inhibition may suppress tumor cell migration in vivo by promoting the upregulation of E-cadherin in the tumor cells. We find that E-cadherin expression appears to be strongly upregulated in the Cl-Amidine treated group compared to the control group (Fig. 7b). This suggests that Cl-Amidine treatment maintained the epithelial-like state of the cells thereby preventing tumor cells from migrating out from the mammary duct.

### BB-cl-Amidine suppresses ductal invasion in an ex vivo model of mammary gland development

**Fig. 8:**
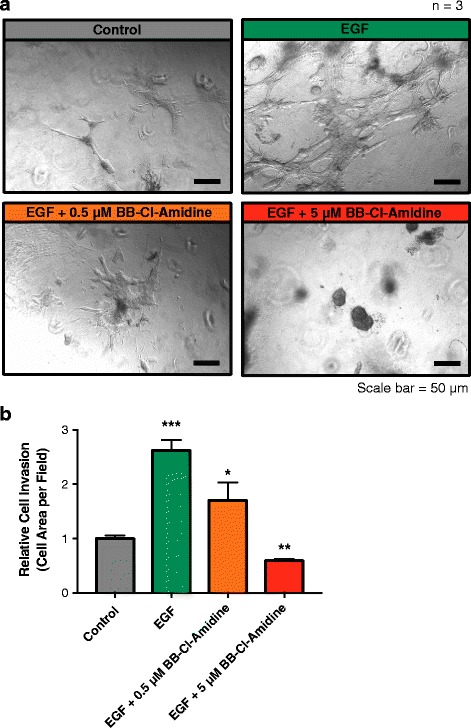
PAD inhibition suppresses EGF-induced ductal invasion and elongation in primary mouse mammary organoids. **a** Isolated mammary gland organoids were cultured in matrigel and were treated with either DMSO (control), EGF (100 ng/ml), EGF (100 ng/ml) + BB-Cl-Amidine (0.5 μM), or EGF (100 ng/ml) + BB-Cl-Amidine (5 μM). Following 6 days of culture, the samples were imaged using Zeiss Axio Observer inverted microscope. **b** The relative area of invasion and elongation (normalized cell area per field) were measured using image J. Samples were normalized to the control. Data were analyzed using unpaired Student’s t-test and are expressed as the mean ± SEM (*p < 0.01; **p < 0.05; ***P < 0.005)

We next utilized a three-dimensional organoid culture system to investigate the effect of PAD2 inhibition on mammary gland morphogenesis*.* We first induced ductal morphogenesis using EGF and found a robust increase in the ductal elongation compared to the control organoid (Fig. 8a and b). We found that when the organoids were treated with 0.5 μM BB-Cl-Amidine, there was a striking reduction in EGF-induced ductal elongation (Fig. 8a and b) with 5 μM BB-Cl-Amidine completely blocking ductal elongation (Fig. 8a and b). These results suggest that PAD2 is required for EGF-induced ductal morphogenesis.

### PAD2 overexpression enhances mammary branching

**Fig. 9:**
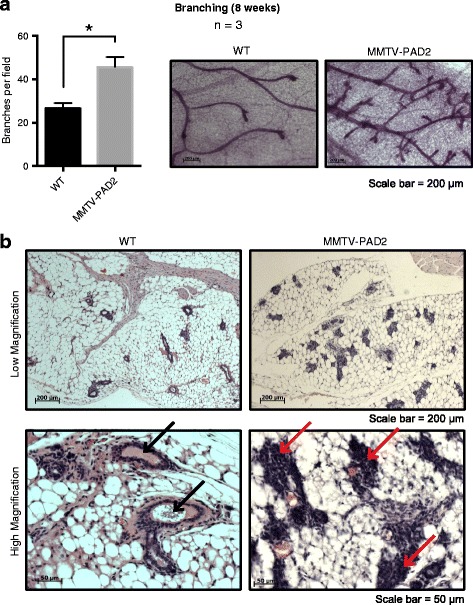
Mammary gland development in diestrus 8 week old female wild type and MMTV-PAD2 mice. **a** Graph quantifying number of branches/field in mammary glands from 8 week old diestrus wild type and MMTV-PAD2 female mice (*n* = 3). Representative whole mount images (20×) of mammary gland development in wild type and PAD2 overexpressing mice. Following isolation, mammary fat pads were stained with carmine-alum for visualization. **b** Images of formalin-fixed-paraffin embedded mammary gland tissue sections following H&E staining at 5× (low magnification) and 20× (high magnification). Note the normal appearance of acini in WT mice showing typical epithelial layer and lumen. In MMTV-PAD2 mice the acini are less organized with apparent infiltration of epithelial cells into the lumen

EGF and its receptor EGFR are known to play critical roles in mammary gland morphogenesis during early development [44–46]. Given the observed links between PAD2 and EGF signaling, we generated a PAD2 overexpressing mice to test whether overexpression of PAD2 in the mammary gland may affect mammary gland development. At 8 weeks of age, we found a 1.7-fold increase in ductal branching in the PAD2 overexpressed mice compared to the wild-type controls (Fig. 9a). Histological evaluation of the mammary tree in adult females revealed that the epithelial cells are hyperplastic in nature with poorly formed ductal structures (Fig. 9b). Together, these data suggest that PAD2 plays an important role in mammary gland morphogenesis during development.

## Discussion

Invasion of the of basement membrane by tumor cells represents a critical step in tumor metastasis. In mouse xenograft models of breast cancer, we have previously shown that systemic treatment with the PAD inhibitor, Cl-Amidine, resulted in increased tumoral basement membrane integrity compared to tumors from mice treated with vehicle alone [14, 47]. In this study, we show that PAD2 depletion or inhibition suppresses tumor cell migration, alters tumor cell morphology, and suppresses the expression of the cytoskeletal regulatory proteins: RhoA, Rac1, and Cdc42. Taken together, these observations support the hypothesis that PAD2 promotes tumor migration and invasion via its role in modulating the tumor cell cytoskeleton.

Interestingly, while performing the cell migration assay, we observed that PAD2-depleted MCF10DCIS cells preferentially adhere to each other, suggesting that PAD2 may be involved in tumor cell migration. In support of this hypothesis, we found that E-cadherin expression is upregulated in MCF10DCIS cells following PAD2 depletion. Previously, we demonstrated that PAD4 plays a role in preventing the EMT and suppressing the invasive potential of breast cancer cells [26], which is in direct contrast to our findings with PAD2. Thus, it appears that there may be a complex interplay between these two family members in breast cancer cells with respect to the EMT and further studies are needed to further tease out this relationship.

Given that EGF signaling plays an important role in tumor cell migration, we tested whether there is a link between PAD2 and EGF. Interestingly, we found that EGF treatment resulted in a strong increase in nuclear PAD2 levels. We have previously shown that nuclear PAD2 can regulate gene expression via citrullination of histone tails at transcription factor binding sites [31, 48, 49]. Taken together, these results suggest that the EGF-induced nuclear targeting of PAD2 may lead to PAD2-mediated upregulation of genes involved in tumor cell migration. In follow up studies, it will be interesting to test whether PAD2-mediated histone citrullination may regulate EGF-induced gene expression.

Since we observed that PAD2 was upregulated after EGF treatment, we then tested whether inhibition of PAD2 can suppress EGF-mediated cell migration. Outcomes from our in vitro and in vivo studies support this prediction as they found that BB-Cl-Amidine suppressed EGF-induced tumor cell migration and invasion. Interestingly, we also observed upregulation of E-cadherin in our PAD inhibitor-treated xenograft model. In addition to links between PAD2 and EGF-mediated tumor cell migration, we also found that overexpression of PAD2 in FVB mice lead to mammary gland hyperbranching, thus supporting a role for PAD2 in mammary tumorigenesis. Taken together, these findings implicate PAD2 in EGF-mediated mammary gland development and tumor cell migration.

From a clinical perspective, many patients with DCIS are either under- or over- treated because clinicians lack the appropriate molecular markers to help predict recurrence or progression. Our finding that PAD2 appears to function within the EGF signaling pathway to modulate DCIS tumor cell migration suggests that PAD2 and/or citrullination may have predictive power for identifying aggressive EGFR+ DCIS lesions. Our finding that the PAD inhibitor, BB-Cl-Amidine, suppresses EGF-induced tumor cell migration, raises the possibility that PAD inhibitors could function in therapeutic formulations for treating EGFR-overexpressing breast cancers. Additionally, it is also possible that PAD inhibitors may synergize with EGFR inhibitors to suppress tumor growth. Future studies are now being planned to test these hypotheses.

## Conclusion

**Fig. 10:**
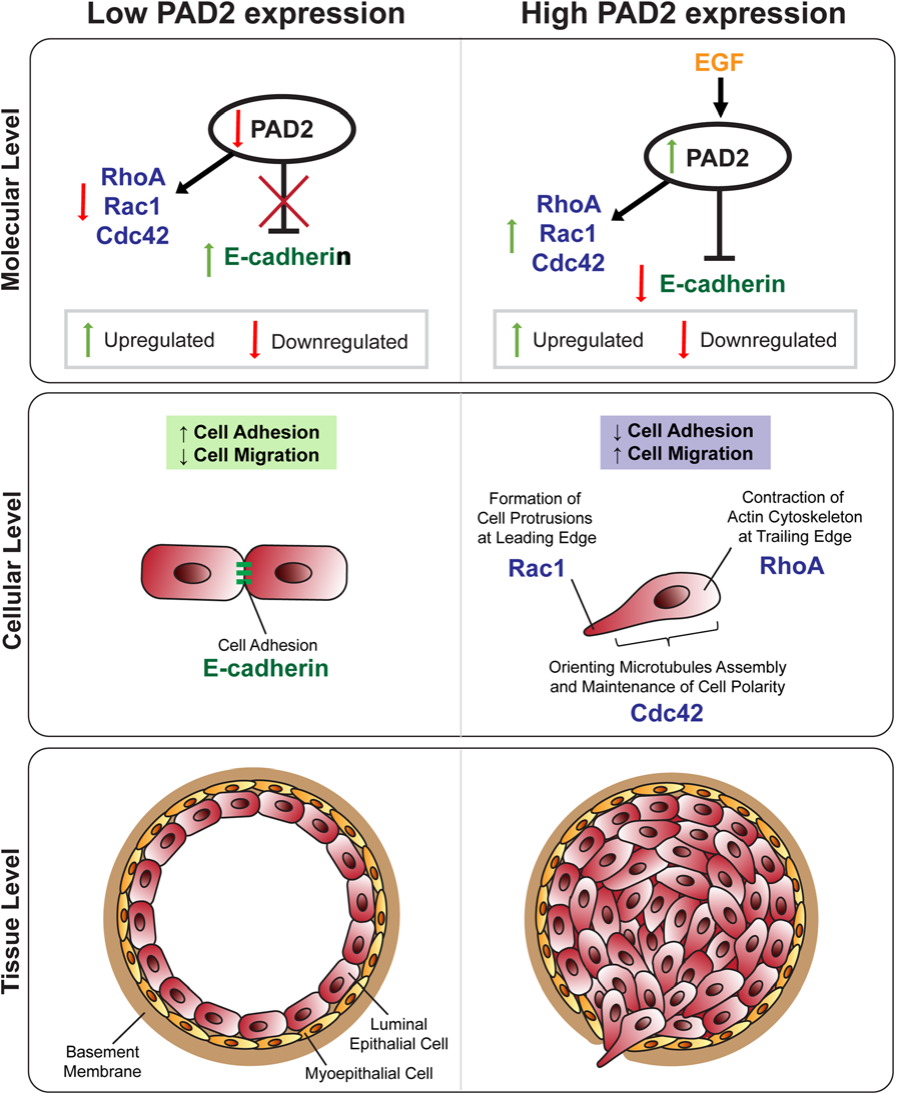
Schematic diagram illustrating the proposed mechanism of PAD2 in mammary carcinoma cell migration. **a** In low PAD2 expressing cells, E-cadherin is upregulated while the Rho family of small GTPases are downregulated, resulting in increased cell-cell adhesion. On the other hand, in high PAD2 expressing cells, E-cadherin is downregulated while Rho family of small GTPases are upregulated, causing increased cell migration. At the tissue level, this increased cell mobility may result in increased cell invasion similar to that observed in invasive mammary carcinoma

In this study, we performed in vitro and in vivo assays to test the involvement of PAD2 in the migration of mammary tumor cells (Fig. 10a). We found that PAD2 depletion or inhibition greatly impairs cell migration and alters the morphology of MCF10DCIS.com cells and that PAD2 overexpression in MCF10AT cells promotes cell migration. In addition, we found that PAD2 depletion suppresses the expression of Rho GTPases (RhoA, Rac1, and Cdc42), which are known to be involved in actin-mediated cell migration. Furthermore, we observed that PAD2 depletion promotes a mesenchymal to epithelial-like transition in MCF10DCIS.com cells, with a robust increase seen in the adhesion marker, E-cadherin. In vivo, we found that treatment of MCF10DCIS.com xenograft tumors with PAD inhibitors upregulate E-cadherin expression and that EGF-induced cell migration upregulates PAD2 expression and activity. We also found that the PAD inhibitor, BB-Cl-Amidine, inhibits EGF-induced tumor cell migration. When then observed that PAD2 overexpression in female FVB mice promotes mammary hyperbranching and enhanced infiltration of epithelial cells into the alveoli of the mammary gland, thus supporting the role of PAD2 in mammary development and tumorigenesis. Furthermore, our mouse mammary organoid studies showed that we can enhance ductal invasion via the use of EGF and prevent invasion using the PAD inhibitor, BB-Cl-Amidine. This suggests that PAD2 likely functions within EGF signaling pathway to mediate cell migration and invasion. Together, these findings suggest that PAD2 plays an important role in mammary tumorigenesis via its role as a mediator of EGF signaling.

## Additional files

Additional file 1: Fig. S2. PAD2 overexpression increases the migratory potential of MCF10AT cells. Relative average areas of wound closure from the wound healing assays (*n* = 6) were calculated using ImageJ and normalized to empty vector controls (* *p* < 0.05). (PDF 690 kb)

Additional file 2: Fig. S1. Depletion of PAD2 suppresses the ability of MCF10DCIS.com cells to form foci. Representative image of crystal violet stained MCF10DCIS.com scrambled-shRNA and *PADI2-*shRNA cells grown for 1-week. (PDF 3631 kb)

Additional file 3: Fig. S3. EGF-induced cell migration is inhibited by BB-Cl-Amidine. Wound healing assays were performed on MCF10DCIS.com scrambled-shRNA and *PADI2*-shRNA cells treated with EGF or BB-Cl-Amidine. The cells were fixed using 4% paraformaldehyde for 32 h after striking the wound. The cells were then visualized and imaged using light microscopy to determine the extent of the wound closure. (PDF 15582 kb)

Additional file 4: Supplemental Methods. (DOCX 15 kb)

## Abbreviations

DCIS: ductal carcinoma in situ
EGF: epidermal growth factor
EMT: epithelial-to-mesenchymal transition
ECM: extracellular matrix
PAD2 or *PADI2*: Peptidylarginine deiminase 2
