# Supplementary figures and images for: Role of peptidylarginine deiminase 2 (PAD2) in mammary carcinoma cell migration
Source: BMC Cancer. 2017 May 26;17:378. doi: 10.1186/s12885-017-3354-x (PMC5446677; doi:10.1186/s12885-017-3354-x)

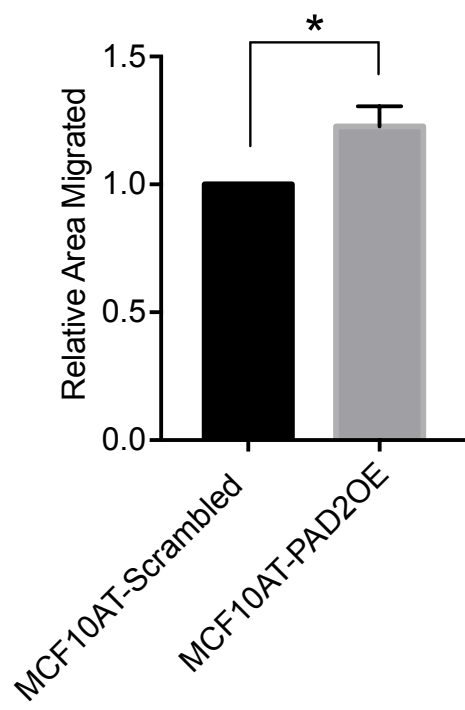

Supplement: Supplementary file 1 — PAD2 overexpression increases the migratory potential of MCF10AT cells. Relative average areas of wound closure from the wound healing assays (n = 6) were calculated using ImageJ and normalized to empty vector controls (* p < 0.05). (PDF 690 kb) [file 12885_2017_3354_MOESM1_ESM.pdf]

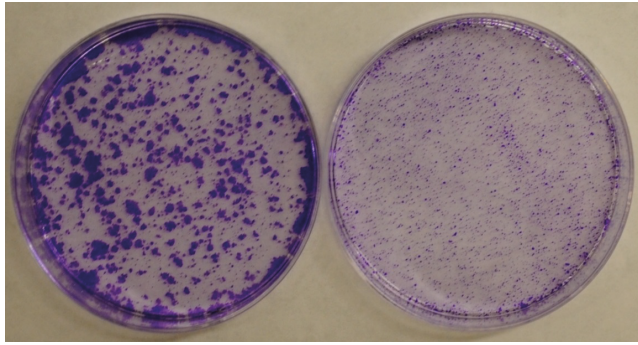

Scrambled  
shRNA

*PADI2*  
shRNA

Supplement: Supplementary file 2 — Depletion of PAD2 suppresses the ability of MCF10DCIS.com cells to form foci. Representative image of crystal violet stained MCF10DCIS.com scrambled-shRNA and PADI2-shRNA cells grown for 1-week. (PDF 3631 kb) [file 12885_2017_3354_MOESM2_ESM.pdf]

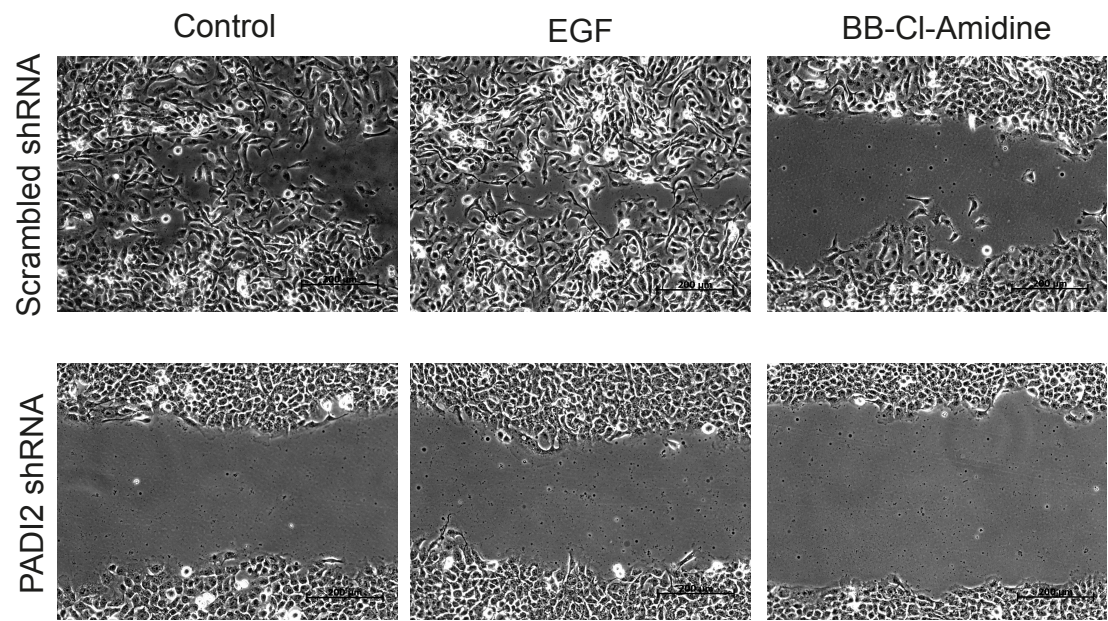

Supplement: Supplementary file 3 — EGF-induced cell migration is inhibited by BB-Cl-Amidine. Wound healing assays were performed on MCF10DCIS.com scrambled-shRNA and PADI2-shRNA cells treated with EGF or BB-Cl-Amidine. The cells were fixed using 4% paraformaldehyde for 32 h after striking the wound. The cells were then visualized and imaged using light microscopy to determine the extent of the wound closure. (PDF 15582 kb) [file 12885_2017_3354_MOESM3_ESM.pdf]
