# Supplementary material for: Role of peptidylarginine deiminase 2 (PAD2) in mammary carcinoma cell migration
Source: BMC Cancer. 2017 May 26;17:378. doi: 10.1186/s12885-017-3354-x (PMC5446677; doi:10.1186/s12885-017-3354-x)
Supplement: Supplementary file 4 — Supplemental Methods. (DOCX 15 kb) [file 12885_2017_3354_MOESM4_ESM.docx]

**Supplemental Methods**

**Focus formation**

2.5 x 10^4^ MCF10DCIS.com cells stably expressing scrambled or *PADI-*shRNA were grown for 1-week in 6 mm dish. After 1-week, the cells were fixed with 4% paraformaldehyde, and were stained with 0.5% crystal violet staining solution for 10 minutes. Three biological replicates were performed.

**Generation of PAD2 overexpressing MCF10AT cells**

To generate lentiviral transduced MCF10AT cells, the PAD2 open reading frame was cloned into a pLenti-PGK-GFP-Puro plasmid (Addgene #19070) as previously described [38]. In brief, the pLenti-PAD2 plasmid or the empty vector pLenti control was packaged into lentiviruses using lentiviral packaging/envelope vectors (pLP1-pMDLg/pRRE, Addgene #12251; pLP2-pRSV-Rev, Addgene #12253; and pVSV-G-pMD2.G, Addgene #12259) and stably transduced MCF10AT cells were selected for in 1 µg/ml puromycin.

**Cell Migration (scratch or wound healing assays)**

**PAD2 depletion**. MCF10DCIS.com cells expressing scrambled shRNA or *PADI2*-shRNA were initially grown in their regular selection media for plating but later serum starved for 24 hours before treatment. Once the cells were confluent, a wound was struck and the detached cells were removed. DMEM/F-12 containing the following treatments were added: control (DMSO), EGF (100 ng/ml), or BB-Cl-Amidine (1 μM). After 32 hours, the cells were fixed for 12 minutes with 4% paraformaldehyde, washed with PBS, and visualized by light microscopy. Three biological replicates were performed.

**PAD2 overexpression**. PAD2-overexpressing or control MCF10AT cells were initially grown in their regular culture media to confluency. Once the cells were confluent, a wound was struck and the detached cells were removed. After 24 hours, the cells were fixed for 12 minutes with 4% paraformaldehyde, washed with PBS, and visualized by light microscopy. Six biological replicates were performed.
